# Implications for Social Support on Prolonged Sleep Difficulties among a Disaster-Affected Population: Second Report from a Cross-Sectional Survey in Ishinomaki, Japan

**DOI:** 10.1371/journal.pone.0130615

**Published:** 2015-06-18

**Authors:** Shoko Matsumoto, Kazue Yamaoka, Machiko Inoue, Mariko Inoue, Shinsuke Muto, Teikyo Ishinomaki Research Group

**Affiliations:** 1 Graduate School of Public Health, Teikyo University, Tokyo, Japan; 2 Department of Family and Community Medicine, Hamamatsu University School of Medicine, Shizuoka, Japan; 3 Department of Hygiene and Public Health, Teikyo University School of Medicine, Tokyo, Japan; 4 Health and Life Revival Council in the Ishinomaki District (RCI), Miyagi, Japan; Chiba University Center for Forensic Mental Health, JAPAN

## Abstract

**Study Objectives:**

This study aimed to investigate the role of social factors, especially social support for sleep, among victims living at home around 1–2 years after the Great East Japan Earthquake and tsunami.

**Design:**

A cross-sectional household survey was conducted between May and December 2012 (14–21 months after the disaster) in the Ishinomaki area, Japan. Univariate and multivariate logistic regression models were used to examine the association between social factors, including social support, and prolonged sleep difficulties (persisting over 1 month). Social support was divided into three functions: emotional, informational, and instrumental support.

**Participants:**

Data were obtained on 2,593 individuals who were living at home after the disaster.

**Results:**

The prevalence of prolonged sleep difficulties was 6.9% (5.8% male, 7.7% female). This study showed that lack of social support has a stronger association with prolonged sleep difficulties than non-modifiable or hardly modifiable consequences caused directly by the disaster, i.e., severity of home damage, change in family structure and income. Among the three dimensions of social support, lack of emotional support showed the strongest association with prolonged sleep difficulties.

**Conclusions:**

Social support, especially emotional support, may positively affect sleep among victims living at home around 1–2 years after a disaster.

## Introduction

Sleep difficulties frequently begin as stress-related phenomena including natural disaster [1,2] and may take a chronic course, persisting for 1 to several years [3,4]. Sleep difficulties are related to physical illness (e.g., cardiovascular dysfunction [5] and other chronic diseases [6]) and psychological illness (post-traumatic stress disorder [7,8] and other mental disorders [9–11]), and they could also bear an influence on economy in disaster-affected societies [12,13]. Therefore, the consequences of sleep difficulties are considerable, especially after disaster.

We have previously investigated the association between social factors and sleep difficulties among survivors of the 2011 Great East Japan Earthquake and tsunami in the Ishinomaki area [14]. That area was among those suffering the greatest devastation by the tsunami along the Pacific coast in the Tohoku region. In that study, the subjects consisted of 4,176 victims who chose to continue living in their damaged homes rather than moving to temporary shelters or housing in the 6–12 months after the disaster. Among those subjects, we found two social factors related to sleep difficulties: the lack of pleasure in life and the lack of relatively strong neighborhood networks. We regarded the lack of pleasure in life as a result of the absence of social networks. Those factors appeared to have stronger associations with sleep difficulties than nonmodifiable or hardly modifiable consequences of the disaster, e.g., housing damage, changes in family structure, and changes in work status.

The quality and quantity of social networks and sleep may change following a disaster. To investigate the role of social factors in sleep difficulties at different time points after a disaster, we conducted the current study. This study used data obtained by interviewing victims who were living in their homes 14–21 months after the Great East Japan Earthquake and tsunami; it excluded the subjects who participated in our previous study. To deepen our understanding of how social networks contribute to better sleep among disaster victims, we especially focused in the present study on the role of social support for sleep difficulties. Social support is a function of social networks: it is accessible to individuals through social networks among other individuals, groups, family, and communities [15].

A number of studies have suggested that social support bears an influence on individual health and well-being. Various theoretical pathways have been postulated to account for this association, including protection against psychological stress [16], encouraging healthy behaviors [17–19] and coping ability [20,21], and promoting cooperation with medical regimens [22]. However, the association between social support and sleep difficulties has not been well documented in a disaster-affected population.

The concept of social support includes its structure and function. The structure is accounted for by quantitative network factors, whereas the function involves more qualitative aspects of support. The four most frequently used defining attributes of functional social support are emotional, instrumental, informational, and appraisal [23,24]. Emotional support is associated with sharing life experiences, involving the provision of empathy, love, trust, and care. Instrumental support involves the provision of tangible aid and services that could assist a person in need. Informational support incorporates the provision of advice, suggestions, and information during times of stress. Appraisal support entails the provision of information that is useful for self-evaluation purposes rather than problem-solving purposes. To our knowledge, no study has investigated which functional aspects of social support particularly play a role in sleep in a disaster-affected population.

The aims of the current study were twofold: (1) to verify the findings in our previous study by examining the association between social factors and sleep difficulties among survivors living at home using data obtained 14–21 months after the Great East Japan Earthquake and tsunami; (2) to investigate the role of social support for sleep difficulties after the disaster. To address the limitations in our previous study, we defined the presence of sleep difficulties in the present investigation as those that lasted over 1 month (hereafter, prolonged sleep difficulties) and that were reported only by the subject, not by representatives of the household. In this study, we also evaluated the perception of three of the four dimensions of functional social support (emotional, instrumental, and informational support) rather than the actual receipt of such support.

There is a lack of research on victims who were living at home at different time points after a natural disaster. The present investigation should thus provide important information for strategies or interventions to prevent prolonged sleep difficulties among a disaster-affected population.

## Methods

After the Great East Japan Earthquake and tsunami occurred in March 2011, the Health and Life Revival Council in the Ishinomaki district (RCI) conducted a face-to-face survey using a semi-structured questionnaire in that area between May and December 2012 (14–21 months after the disaster). This took place after the first phase of the survey, which was conducted 6–12 months after the disaster.

As the first phase of the survey was being conducted, the RCI provided various kinds of support to victims who continued living in their homes; this support was based on their needs as revealed in the survey. The support included maintenance of their housing, medical and welfare support, and providing daily necessities. The RCI targeted victims who remained in their homes because such people received less support from governmental agencies than those living in temporary shelters.

The primary objective of the second phase of the survey was to increase the efforts of the RCI by investigating stay-at-home victims who were not at home or who were resident in other parts of the Ishinomaki area at the time of the first survey. The activities of the RCI and the detailed methodology of the first survey can be found elsewhere [14].

The second phase of the survey consisted of three parts. The first was conducted in face-to-face manner by a representative of the household, and it included questions related to household demographics and social background of the household members. The second involved obtaining responses from all household members who were at home at the time of the visit; it mainly included questions related to interviewee lifestyle. The third was in the form of a questionnaire given to each adult household member, and it was returned to the RCI by post upon completion. The items on this third questionnaire mainly related to the physical and psychosocial health condition of the respondents (including prolonged sleep difficulties).

**Fig 1:**
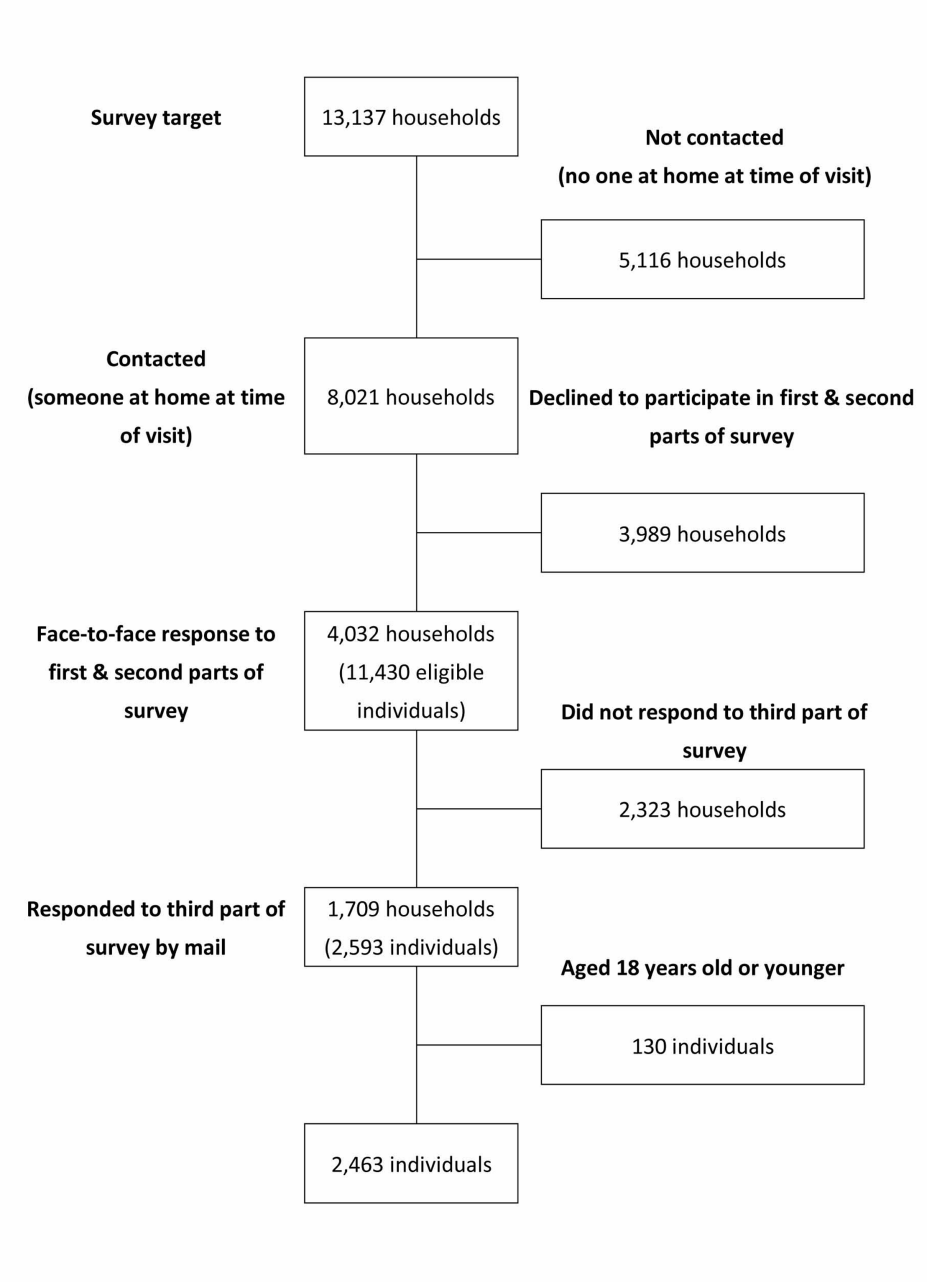
Flow diagram of study participants in the analyses.

The data from the second phase of the survey were used for the present study. Fig 1. shows the flow of the participants through this study. We visited 13,137 households and were able to contact people in 8,021; in the other 5,116 households, no one was at home at the time of the visit. Among the 8,021 households, individuals from 4,032 households (11,430 eligible subjects) responded to the survey, and 2,593 individuals from 1,709 households responded to the third step of the survey. Of those 2,593 subjects, we excluded 130 who were aged 18 years or under because they were not ineligible for the third step of the survey; thus, the data on 2,463 household members were used for our analysis.

### Ethics statement

All the participants gave their written permission to be interviewed. Ethical approval for this study was obtained from the Institutional Review Board of Teikyo University (reference number: 12–079).

### Measurements

#### Prolonged sleep difficulties

With the third questionnaire, the presence of prolonged sleep difficulties was evaluated with the question, “Do you have any subjective symptoms that have lasted for longer than 1 month that may affect your daily life?” Among the 19 possible responses was “sleep difficulties”; other items were “none,” “headache,” “dizziness,” “throbbing heart,” “stomachache,” “loss of appetite,” “overeating,” “asthma,” “sore throat,” “phlegm and coughing,” “blurred vision,” “dermatitis,” “allergy,” “stiff shoulders,” “backache,” “knee pain,” “oversleeping,” and “other.” The respondents could choose as many items as they thought applicable. Respondents who selected “sleep difficulties” were regarded as having prolonged sleep difficulties; others were regarded as not having such difficulties.

#### Demographics

The sex and age of the participants were obtained as basic demographic information. Age was divided into two categories: (1) <65 years; (2) ≥65 years. We used 65 years as the cutoff for the categories because it is the common retirement age in Japan. Age was also treated as a continuous variable; it was analyzed in 10-year units.

#### Social factors

Data on the following were obtained as household sociodemographic information: the number of household members; the source of the household’s income; local government recognition of the severity of damage to the home; and changes in family structure or income due to the disaster. Those were the same as or similar to the parameters used in our previous study, thereby allowing a comparison of the results. The number of household members was divided into three categories: (1) one; (2) two; and (3) more than two. The source of income was divided into the following two categories: (1) only salary or pension and salary; (2) only pension, public livelihood assistance, unemployment allowance, no regular income, or other. The severity of damage to the home was rated on a five-point scale according to the government criteria of house damage: (0) none; (1) partially damaged; (2) half-destroyed; (3) largely destroyed; and (4) totally destroyed. Many participants continued to live in their houses that were rated as being “totally destroyed” even after parts had totally collapsed or were swept away by the tsunami. Change in family structure was defined as any change in the number of household members due to the disaster, e.g., death, relocation of family members. Changes in family structure or income as a result of the disaster were responded to dichotomously.

Social support was divided into its three dimensions: emotional, instrumental, and informational support. We did not assess appraisal support—the other function of social support—in this study; this was due to limited interview time and also because of the relative lack of research and evidence on appraisal support for health compared with other dimensions of social support.

We developed questionnaires related to social support with reference to a social survey called the National Family Research of Japan [25]. That is a series of nationwide surveys of families based on random samples in the country, and it has been conducted since 1998. Emotional support was assessed by means of the following items: having someone to talk to; and having someone to consult about problems. Respondents who gave affirmative replies to either or both items were regarded as having emotional support, and others were regarded as not having such support. Instrumental support was assessed through the following items: having someone who provides physical support or care; and having someone who provides financial support. Respondents who gave affirmative replies to either or both items were regarded as having instrumental support, and others were regarded as not having such support. Informational support was assessed by the following item: having someone who provides information, and the response was yes or no. We also developed a dichotomous variable of social support, in which respondents who had any of three dimensions of social support were regarded as having such support, and those who had none were taken as lacking this support. The dichotomous variable was developed to examine the overall effect of social support regardless of its dimensions considering the overlaps in concepts of social support [23,26,27].

### Statistical analysis

We analyzed the prevalence of prolonged sleep difficulties according to the respondents’ characteristics. Logistic regression analyses were then used to examine the statistical associations between the outcome variable (prolonged sleep difficulties) and the explanatory variables related to demographics and social factors.

In the logistic regression analyses, we first conducted a univariate analysis for each explanatory variable, and we calculated the odds ratios (ORs) with 95% confidence intervals (95% CIs) (Model 1). Next, we developed multivariate models (Model 2), in which social support was rated dichotomously (having any of the three types of social support or having none). To investigate the role of social support in depth, we developed Model 3. With this model, instead of the dichotomous variable related to social support used in Model 2, we employed three variables related to social support, each of which described emotional, instrumental, and informational support. In Models 2 and 3, missing data were excluded from the analyses.

By way of supplementary analyses, we conducted multivariate models using a stepwise method for all variables (inclusion and exclusion criteria = 0.3 for each) to improve statistical power. Finally, to examine the effects of missing data, we developed multivariate models, which included “missing” as a category for each item. Further, we used a multiple imputation method in the multivariate models under the assumption of missing at random [28].

We treated all the variables except age and frequency of leaving the house as categorical variables in the logistic regression analyses. All analyses were performed using SAS software (version 9.3; SAS Institute, Cary, NC, USA). All tests were two-sided, with a significance level of 5%.

## Results

Among the 8,021 households we were able to contact at the time of the visit, 1,709 responded to the survey (response rate, 21.3%). The characteristics of the respondents and the prevalence of prolonged sleep difficulties appear in Table 1. The prevalence of prolonged sleep difficulties was 6.9% (5.8% male, 7.7% female). The mean age (standard deviation) was 57.1 (15.9) years, and 64.3% of respondents were younger than 65. In all, 17.9% of the respondents’ homes were recognized by the local government as being totally destroyed. Moreover, 25.1% of respondents experienced a change in family structure, and 42.0% experienced a change in income through the disaster. The prevalence of prolonged sleep difficulties was especially high among those who had no social support (13.1%). The proportions of missing values were below 5.0%—except for severity of damage to the home (7.0%) and variables related to social support (5.4%). Regarding the three dimensions of social support, the proportion of respondents who reported having instrumental support was much lower than that of those who reported not having such support (9.6% versus 85.0%). This finding was reversed when comparing the proportion of respondents who reported receiving emotional support with those who did not have such support (81.8% versus 12.8%).

**Table 1:** Characteristics of respondents and prevalence of prolonged sleep difficulties.

	*n*	%^a^	Prolonged sleep difficulties (%)^b^
**All**	2463	100.0	6.9
**Sex**			
Male	969	39.3	5.8
Female	1488	60.4	7.7
Missing	6	0.2	0.0
**Age**			
all	[57.1±15.9]^c^		
<65 years	1584	64.3	6.1
≥65 years	879	35.7	8.3
**Number of household members**			
One	178	7.2	9.0
Two	746	30.3	7.0
More than two	1539	62.5	6.6
**Source of household income**			
Salary or pension and salary	1899	77.1	6.9
Only pension and other^d^	564	22.9	7.1
**Severity of house damage**			
Totally destroyed	441	17.9	7.7
Largely destroyed	1186	48.2	7.2
Half-destroyed	110	4.5	8.2
Partially damaged	370	15.0	6.5
None	184	7.5	4.4
Missing/unknown	172	7.0	5.8
**Change in family structure due to disaster**			
Yes	618	25.1	6.6
No	1840	74.7	7.0
Missing	5	0.2	0.0
**Change in income due to disaster**			
Yes	1034	42.0	7.4
No	1416	57.5	6.6
Missing	13	0.5	0.0
**Having any social support**			
Yes	2139	86.9	6.3
No	191	7.8	13.1
Missing	133	5.4	7.5
**Having emotional social support**			
Yes	2015	81.8	6.2
No	315	12.8	11.1
Missing	133	5.4	7.5
**Having instrumental social support**			
Yes	237	9.6	5.9
No	2093	85.0	7.0
Missing	133	5.4	7.5
**Having informational social support**			
Yes	1025	41.6	5.3
No	1305	53.0	8.1
Missing	133	5.4	7.5

Prolonged sleep difficulties: sleep difficulties that lasted over 1 month

*n*: number of respondents

^a^ The number in each category was divided by the total number of participants (2463)

^b^ Prevalence of prolonged sleep difficulties in each category

^c^ [mean ± standard deviation]

^d^ “Other” includes public livelihood assistance, unemployment allowance, and no regular income.

The results of the univariate (Model 1) and multivariate model (Models 2 and 3) are shown in Table 2. In Model 2, being older (OR = 1.02, 95%CI = 1.00–1.03 per 10-year increase in age) and having no social support (OR = 2.55, 95%CI = 1.58–4.11 versus having some social support) were associated with a higher likelihood of prolonged sleep difficulties. The nonmodifiable or hardly modifiable consequences caused directly by the disaster, such as severity of damage to the home and change in family structure or income due to the disaster, were not significantly associated with prolonged sleep difficulties.

**Table 2:** Logistic regression results: odds ratios of prolonged sleep difficulties and 95% confidence intervals.

	Model 1^a^		Model 2^b^		Model 3^c^	
			(n = 2097)		(n = 2097)	
	OR (95%CI)	p-value	OR (95%CI)	p-value	OR (95%CI)	p-value
**Sex**						
Male	1.00		1.00		1.00	
Female	1.35 (0.97–1.88)	0.07	1.40 (0.98–2.01)	0.07	1.49 (1.04–2.14)	0.03
**Age**						
Age (in 10-year increments)^d^	1.14 (1.03–1.27)	0.01	1.02 (1.00–1.03)	0.01	1.02 (1.00–1.03)	0.01
**Number of household members**						
One	1.35 (0.78–2.35)	0.56	1.54 (0.81–2.93)	0.19	1.61 (0.84–3.08)	0.16
Two	1.05 (0.74–1.49)		0.87 (0.57–1.34)		0.88 (0.57–1.35)	
More than two	1.00		1.00		1.00	
**Source of household income**						
Salary/pension and salary	1.00	0.88	1.00	0.16	1.00	0.18
Only pension and other^e^	1.03 (0.71–1.49)		0.70 (0.43–1.15)		0.71 (0.43–1.17)	
**Severity of house damage**						
Totally destroyed	1.86 (0.84–4.10)	0.59	1.69 (0.76–3.79)	0.71	1.74 (0.78–3.91)	0.66
Largely destroyed	1.71 (0.82–3.60)		1.55 (0.73–3.28)		1.53 (0.72–3.25)	
Half-destroyed	1.97 (0.74–5.26)		1.98 (0.73–5.34)		2.04 (0.75–5.53)	
Partially damaged	1.54 (0.68–3.50)		1.57(0.69–3.61)		1.57 (0.69–3.61)	
None	1.00		1.00		1.00	
**Change in family structure due to disaster**						
Yes	0.94 (0.65–1.35)	0.72	0.85 (0.56–1.29)	0.45	0.84 (0.55–1.27)	0.41
No	1.00		1.00		1.00	
**Change in income due to disaster**						
Yes	1.13 (0.82–1.54)	0.46	1.17 (0.83–1.66)	0.38	1.17 (0.82–1.66)	0.38
No	1.00		1.00		1.00	
**Having any social support**						
Yes	1.00		1.00			
No	2.29 (1.45–3.61)	<.001	2.55 (1.58–4.11)	<.001		
**Having emotional social support**						
Yes	1.00				1.00	
No	1.91 (1.28–2.83)	<.01			2.05 (1.35–3.11)	<.001
**Having instrumental social support**						
Yes	1.00				1.00	
No	1.21 (0.69–2.12)	0.52			1.37 (0.72–2.60)	0.34
**Having informational social support**						
Yes	1.00				1.00	
No	1.61 (1.15–2.26)	0.01			1.55 (1.08–2.21)	0.02

95%CI, 95% confidence interval; OR, odds ratio

^a^ Model 1, univariate model

^b^ Model 2, multivariate model in which social support was rated dichotomously (having any social support or not)

^c^ Model 3, multivariate model in which social support was categorized into three dimensions

^d^ The odds ratio reflects the change in the odds of sleep difficulties per 10-year increase in age.

^e^ “Other” includes public livelihood assistance, unemployment allowance, and no regular income

Instead of the broader indicator related to social support used in Model 2 (having any kind of social support versus having no social support), three dimensions of social support (emotional, instrumental, and informational support) were used as explanatory variables in Model 3. In this model, not having emotional social support (OR = 2.05, 95% CI = 1.35–3.11 versus having emotional social support) and not having informational support (OR = 1.55, 95% CI = 1.08–2.21 versus having informational social support) were associated with a higher likelihood of prolonged sleep difficulties; however, not having instrumental support did not show statistical significance. Loss of any of the three dimensions of social support showed the same direction of effect for prolonged sleep difficulties—even though the proportions of respondents who reported having any of the social support dimensions versus having none were quite different.

With the stepwise method applied to Models 2 and 3 as supplementary analyses, there was no change in the direction of effect and statistical significance for any of the explanatory variables. Further, when “missing” was treated as one category for each item in Models 2 and 3 and the multiple imputation method was applied to these models, the results did not largely differ from the models that excluded the missing (data not shown).

## Discussion

We examined the social factors associated with prolonged sleep difficulties among victims who were living at home 14–21 months after the Great East Japan Earthquake and tsunami. Lack of social support showed a stronger association with sleep difficulties than nonmodifiable or hardly modifiable consequences caused directly by the disaster (e.g., severity of house damage and change in family structure and income due to the disaster). Among the three dimensions of social support, lack of emotional support in Model 3 showed the strongest association with prolonged sleep difficulties (*p* = <.001).

### Prevalence of sleep difficulties

The prevalence of prolonged sleep difficulties observed in the present study was much lower than that in our previous study, where the prevalence of sleep difficulties was 15.0% (9.2% male, 20.2% female). One reason for the difference may be dissimilarity in the age of the study participants. The mean age of the respondents was 65.5 years in our previous study, which used data from the first phase of the survey; the mean age was 57.1 years in the present study. Because age was not statistically associated with sleep difficulties in the previous study and it was found to be only weakly associated in the present one (OR = 1.02 per 10-year increase in age in multivariate models), we believe the influence of age to be limited. In addition, this huge disaster had a tremendous impact on population movement, which may have affected the characteristics of the study participants. However, based on a demographic report released by the city of Ishinomaki, the greatest population movement occurred within 6 months after the disaster [29]. Thus, the effect of population movement on the characteristics of the study participants would appear to have been limited.

Further, the discrepancy in the definition of sleep difficulties could be another reason. In our previous study, we evaluated sleep difficulties by asking whether respondents had sleep problems at the time of interview. In the present study, we focused on prolonged sleep difficulties that had lasted for over 1 month, which does not allow a simple comparison between the two studies. According to one international survey, which assessed the prevalence of sleep difficulties (difficulties in initiating or maintaining asleep) that had lasted over 2 weeks, the prevalence was 5.7%–6.3% among the general population in Japan [30]. The prevalence of sleep difficulties was somewhat higher than in the present study, though the difference was not great. Considering that sleep disturbances are part of the normal response to trauma and could diminish with time, the improvement in the prevalence of sleep difficulties observed in the present survey could be because the disaster victims were recovering from such disaster-triggered difficulties 1–2 years after the event.

A study of the child survivors of the Great East Japan Earthquake and tsunami living in Ishinomaki found only a low correlation between sleep duration and traumatic symptoms 8 months after the disaster [31]. However, other studies of adult or adolescent victims of natural disaster found significant associations between sleep difficulties and traumatic symptoms including psychological or psychosomatic symptoms, and those associations could be maintained over time after the disaster [32–34]. Based on their findings, survivors with persistent sleep difficulties might be at greater risk of psychiatric disorders and warrants special attention. Further research is required to determine this interpretation.

### Social factors associated with sleep difficulties

Lack of social support showed a stronger association with sleep difficulties than nonmodifiable or hardly modifiable consequences caused directly by the disaster. These findings are similar to those from our first-phase survey, which was conducted 6–12 months after the earthquake. The lack of association between nonmodifiable or hardly modifiable consequences caused directly by the disaster and sleep difficulties found in the present study may be because of the time interval between the disaster and the survey. The results of this study suggest another possible explanation: the positive effect of social support on sleep could override the negative association between sleep difficulties and the direct consequences of the disaster. Further research is needed to examine this interpretation.

Regarding the severity of house damage, people living in half-destroyed houses in Models 2 and 3 were more likely to have sleep difficulties than individuals living in totally destroyed or largely destroyed houses; however, the differences were not statistically significant. One reason could be that people living in totally destroyed or largely destroyed houses were able to receive considerable financial support from the government (i.e., grant for reconstruction of houses, loan for rebuilding of their lives, or tax reduction). Individuals living in half-destroyed houses received much less. During the second phase of the survey, we heard of complaints related to inappropriate evaluations of house damage and a sense of unfairness related to governmental support depending on the judgment of severity of house damage. The gap in governmental support between residents in totally or largely destroyed houses and those in half-destroyed houses may have been reflected in the higher likelihood of sleep difficulties among people living in half-destroyed houses.

### Role of social support

Two theoretical models explain the process by which social support promotes well-being and health: the main-effect model and the stress-buffering model [35]. According to the main-effect hypothesis, social support is beneficial—irrespective of whether or not individuals are under stress. Regardless of stress level, both perceived support and social integration influence well-being. By contrast, the stress-buffering model postulates that in the face of psychological stress, individuals may be protected against developing stress-related outcomes through strong social support—in contrast to individuals with weak support. We believe that these two models may both be used to interpret our findings. Perceiving that other people are present or will be present could contribute to survivors’ well-being by promoting a sense of belonging or being cared for. In addition to protection against stress, loneliness, and the sense of isolation caused by the disaster, this sense of belonging and being cared for could lead to better sleep.

Disaster is a well-known contributor to poor physical and mental health. This could be attributable to the changes in the quality and quantity of social support following a disaster [36]. At the time of the survey in the present study (14–21 months after the disaster), the survivors were mostly provided with the daily necessities of life. However, according to a report by volunteer centers established by the National Council of Social Welfare, the number of volunteers those centers dispatched to disaster-affected areas dramatically decreased during the first year after the disaster [37]. Many survivors also had to deal with relocation of their neighbors. Further, the survivors felt irritation and dissatisfaction with the delayed recovery of their normal lives or their communities, and they had a sense of anxiety or insecurity about the future. These experiences and feelings could undermine perceptions of social support. This could cause psychological distress and prolonged sleep difficulties as a result.

Among the three dimensions of social support, 81.8% of respondents reported having emotional social support, whereas only 9.6% reported having instrumental support. Although we could not determine the reason for this difference, one possibility is that disaster-affected victims seek emotional support rather than tangible forms of support more than 1 year after the event. However, we found the same direction of effect with lack of the three dimensions of social support for prolonged sleep difficulties: in Model 3, emotional social support showed the strongest association with prolonged sleep difficulties. This is consistent with previous findings, and it suggests that emotional support is the most important type of social support for health and well-being in both general [26,38–40] and disaster-affected populations [41]. To promote the effectiveness of emotional support in a disaster context, the type of social support provided has to be appropriate for the needs or challenges of a particular stressful event. According to one hypothesis, informational and tangible support are more effective for controllable events, whereas emotional support is more effective for uncontrollable events [42].

### Limitations

To our knowledge, this is the first study to investigate the role of social support for sleep difficulties in a disaster-affected population. However, there are several limitations with this study. First, we adopted a cross-sectional design, which limits the causal inferences for the associations found. Moreover, the lack of information on sleep difficulties prior to the disaster prevented controlling for pre-disaster sleep difficulties in our analyses.

Second, the survey participants were selected by a nonrandom method. Compared with the demographic characteristics of Ishinomaki residents at the time of the second phase of the survey [29], our study included a greater proportion of older and female participants. Moreover, compared with damages in residents reported by Ishinomaki city [43], we included a greater proportion of participants whose houses were severely damaged. This could be because our survey aimed to provide support to the survivors living at home. Furthermore, the prevalence of sleep difficulties may have differed between people who responded to our survey and those who did not.

Third, we were unable to evaluate the severity or frequency of sleep difficulties; instead, we simply asked whether the participants had suffered from sleep difficulties for longer than 1 month. Furthermore, regarding social support, we did not assess the duration and amount of social support received by victims, but we just asked whether they perceived social support at the time of survey. This information should be important for a rigorous evaluation of the role of social support on sleep. We were unable to evaluate the validity of the social support questionnaires we used before starting the survey owing to a lack of time and human resources. However, by employing questionnaires that had already been used in relatively large surveys over a long period, we believe that the validity was warranted to some extent. In measuring each dimension of social support, our assessment used quite similar items to other well-established instruments employed in social support surveys, e.g., the Medical Outcomes Study Social Support Survey [44].

Fourth, our study included only victims who were living at home after the disaster, not those living in temporary shelters; that may have limited the generalizability of our findings. However, one study, which investigated the effect of social support on mental health among victims of the Great East Japan Earthquake and tsunami who were living in temporary shelters, also reported that participants without social support were at risk of psychological distress [45]. Although we did not use the same outcome variable, that study may support our findings if applied to victims living in temporary shelters.

Fifth, regarding the variables “change in family structure due to disaster” and “change in income due to disaster,” we did not determine whether the number of household members or income had increased or decreased as a result of the disaster. In light of the situation, we assume that most of such changes would have been decreases, though future investigations should address this issue.

Finally, missing data may have affected our results. However, because we obtained similar results in our supplementary analyses using the multiple imputation method, it would appear that the effects of missing data on our findings were limited.

Despite its limitations, this study provides important implications for future studies. Improving social support among victims in disaster-affected communities would appear to be an important step in promoting health and recovery in such communities. It is necessary to deepen our understanding of ways to reinforce social support among survivors.
